# What Is the Evidence for “Food Addiction?” A Systematic Review

**DOI:** 10.3390/nu10040477

**Published:** 2018-04-12

**Authors:** Eliza L. Gordon, Aviva H. Ariel-Donges, Viviana Bauman, Lisa J. Merlo

**Affiliations:** 1 Department of Clinical and Health Psychology, University of Florida, 1225 Center Drive, Gainesville, FL 32603, USA; ahariel@ufl.edu (A.H.A.-D.); baumanv@ufl.edu (V.B.); 2 Center for Addiction Research and Education, Department of Psychiatry, University of Florida, 1149 Newell Drive, Gainesville, FL 32610, USA; lmerlo@ufl.edu

**Keywords:** food addiction, eating behavior, process addiction, systematic review

## Abstract

The diagnostic construct of “food addiction” is a highly controversial subject. The current systematic review is the first to evaluate empirical studies examining the construct of “food addiction” in humans and animals. Studies were included if they were quantitative, peer-reviewed, and in the English language. The 52 identified studies (35 articles) were qualitatively assessed to determine the extent to which their findings indicated the following addiction characteristics in relation to food: brain reward dysfunction, preoccupation, risky use, impaired control, tolerance/withdrawal, social impairment, chronicity, and relapse. Each pre-defined criterion was supported by at least one study. Brain reward dysfunction and impaired control were supported by the largest number of studies (*n* = 21 and *n* = 12, respectively); whereas risky use was supported by the fewest (*n* = 1). Overall, findings support food addiction as a unique construct consistent with criteria for other substance use disorder diagnoses. The evidence further suggests that certain foods, particularly processed foods with added sweeteners and fats, demonstrate the greatest addictive potential. Though both behavioral and substance-related factors are implicated in the addictive process, symptoms appear to better fit criteria for substance use disorder than behavioral addiction. Future research should explore social/role impairment, preoccupation, and risky use associated with food addiction and evaluate potential interventions for prevention and treatment.

## 1. Introduction

The term “addiction” is commonplace in present society, despite the lack of consensus on an established clinical definition (see Table 1 for definitions set forth by various health-related professional organizations). In clinical practice, there is no official diagnosis of “addiction.” Instead, the Diagnostic and Statistical Manual of Mental Disorders, Fifth Edition (DSM-5) states that the term may be used to describe severe substance use disorders [1].

Criteria for substance use disorder diagnoses include 11 biopsychosocial symptoms grouped into four categories (see Table 2) [1]. Diagnostic criteria focus on the consequences (e.g., symptoms, distress, and impairment in daily functioning) of addictive disorders, however, research has provided insight into the actual process (e.g., the neurobiological correlates) of addiction. Some important findings focus on neurological factors related to reward and motivation, including DeltaFosB (∆FosB; a gene transcription factor), dopamine, and opioid expression [2,3,4].

Behavioral and biological indicators of addiction have also been observed in certain excessive behaviors [1], and research highlighting these similarities has sparked interest in addictive behavior more generally [5]. The proposed “behavioral addictions” reflect dependence on a behavior or feeling brought about by an action, as opposed to a substance [6]. Several studies have confirmed similarities between behavioral and substance addictions regarding ∆FosB, dopamine, and opioid expression; impaired control over the behavior; neglect of relationships and role obligations; and continued problematic behavior in the face of negative health outcomes [5,7]. As a result, the DSM-5 recently introduced a new diagnostic category, Non-Substance-Related Disorders, within the newly-named Substance-Related and Addictive Disorders section of the manual. This category currently includes only gambling disorder, however, several other behaviors were considered, including compulsive overeating [5,8], problematic sexual behavior [9], and excessive Internet gaming [5,9]. Although overeating was ultimately excluded from this category due to insufficient empirical evidence, discussion regarding the addictive potential of food has continued. Indeed, organizations such as the American Society of Addiction Medicine (ASAM) have chosen to include “food addiction” in their list of possible addictive disorders [10], and a number of studies have observed clear biological and behavioral similarities between drug use and overeating (i.e., altered dopamine expression, cravings, relapse to highly palatable food) [11,12,13]. In a review of food addiction studies in humans, Meule and Gearhardt [14] reported that four out of the 11 DSM-5 substance use disorder symptoms were empirically supported by studies of highly palatable food, and that the remaining seven symptoms were “plausible” based on the literature available at that time. Several additional studies on food addiction have been published since that review.

Nonetheless, there have been inconsistencies regarding the definition of food addiction. A variety of approaches have been used to measure it, such as self-report questionnaires [18]; patient self-identification [19]; and the Yale Food Addiction Scale (YFAS), which is currently the best available measure for evaluating food addiction based upon modified DSM criteria for substance use disorders [20,21]. Some have erroneously conceptualized food addiction as either obesity or binge eating [22,23,24,25], yet mounting evidence indicates that these constructs are distinct [26,27]. Though some individuals with obesity may display neurological and behavioral similarities to individuals addicted to drugs [24], estimates suggest that only approximately 24.9% of overweight/obese individuals report clinically-significant symptoms of food addiction and 11.1% of healthy-weight individuals also report these symptoms [28]. Similarly, while food addiction symptoms are associated with binge eating behavior and account for 6–14.8% of the unique variance in binge eating disorder [28], current estimates suggest that only approximately 56.8% of individuals with binge eating disorder report clinically significant food addiction symptoms [29]. Although there is substantial overlap between food addiction and binge eating symptoms, the two constructs are not synonymous [26,27].

The concept of food addiction remains controversial [25,30,31]. Some researchers question whether food or eating can be addictive if it is necessary to our survival [25], while others point out the common biological (e.g., brain reward pathways, ΔFosB expression), behavioral (e.g., relapse, using more than intended), and psychological (e.g., preoccupation, impaired control) similarities between the compulsive consumption of highly palatable foods and use of addictive drugs [2,32,33]. Nevertheless, critics and proponents alike agree that more research is needed to confirm the validity of food addiction [30,34]. A non-systematic review by Hone-Blanchet and Fecteau [31] comparing animal and human models of food addiction to characteristics of substance use disorder concluded that there was significant overlap between the two conditions, but that more research was needed. Extant published systematic reviews on the concept of food addiction have either conflated obesity with food addiction or excluded animal studies [22,28,29]. As such, a more recent and inclusive systematic review was needed. The present systematic review aimed to summarize the peer-reviewed empirical literature examining the evidence for food addiction in both animal and human studies. The chosen method involved assessing its association with key characteristics of addiction in relation to food: (a) neurobiological changes, (b) preoccupation with the substance, (c) impaired control, (d) social impairments, (e) risky use, (f) tolerance/withdrawal, (g) chronicity of the condition, and (h) relapse [1,10,17].

## 2. Materials and Methods

Data collection, review, reporting, and discussion were conducted according to the Preferred Reporting Items for Systematic Reviews and Meta-Analyses (PRISMA) Statement [35,36]. The literature search was carried out in PubMed and PsychINFO databases using varying combinations of the following keywords: food addiction, addiction, process addiction, binge eating, hedonic eating, compulsive overeating, compulsive eating, eating behavior, food, eat, feeding behavior/psychology, food preferences, food habits, hyperphagia, eating disorders, obesity, overeat*. Meshterms were used in the PubMed search. Filters were used in both databases according to the study’s predetermined inclusion and exclusion criteria. Given that the “study type” filters on PubMed only identified articles in print, a second search was done using the same search terms without filters in order to identify recent articles published online before print. Additional studies were identified through review of the references listed in the identified articles. Due to the proliferative nature of research on food addiction, two searches were done: the first was completed on 29 June 2016, and the second was completed on 8 January 2018. Protocols were followed for both searches exactly as described above, with the exception that the second search included only articles published since 30 June 2016.

Articles were included if their stated purpose was to test the validity of the food addiction construct, and if they fulfilled the following modified PICOS (Participants, Interventions, Comparisons, Outcomes, and Study Design) criteria [35,36]. Acceptable participants included humans or animals of any age with no specific limitations on disease or diagnosis. Only quantitative, empirical, peer-reviewed studies published in the English language were included. The American Psychological Association’s defines “quantitative” studies as those which “provide numerical representation of observations for the purpose of describing and explaining the phenomenon studied followed by the application of…statistical methods” [37], (“Quantitative Study”). Therefore, studies using self-report measures that produced a numerical outcome (e.g., Likert scale, yes/no, hunger ratings) were considered quantitative. Empirical studies were defined as those “based on…systematic observation, or experiment, rather than theory or general philosophical principle” [37] (“Empirical Study”). Studies examining any type of intervention or comparison (e.g., randomized control trial, cross-sectional) within these constraints were included in order to accurately reflect the heterogeneous nature of the existing literature. Studies could be published online or in print, and no limits were set on date of publication. Finally, because not all overweight/obese individuals or individuals with eating disorders report addiction-like symptoms related to food [29], studies defining food addiction solely by weight, BMI, or eating disorder diagnosis were excluded.

**Figure 1:**
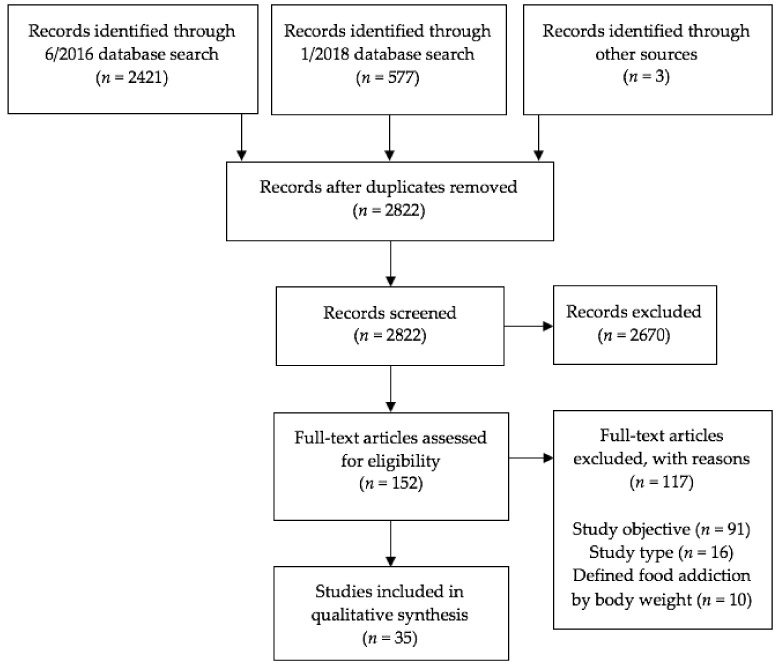
Study selection flow diagram, presented according to the Preferred Reporting Items for Systematic Reviews and Meta-Analyses (PRISMA) statement [35,36].

Articles selected from PubMed and PsychINFO were reviewed first by title, then abstract, and finally full article for relevancy and eligibility using the inclusion criteria described above. The first author independently assessed study eligibility, and articles whose eligibility was unclear were reviewed by two experienced obesity researchers. The last included study was identified in January 2018. A flowchart for study inclusion is depicted in Figure 1.

The data extraction form used for this study was modeled after forms used in similar systematic reviews (e.g., [28]) and was modified for the current paper. The following data were extracted: author/year of publication, study type, sample characteristics, number of subjects in treatment/control groups, main independent variable(s), and main outcome variable(s). Two of the current authors assessed risk of bias on the study level using a modified combined scoring system based on those reported by Jamaty and colleagues [38] and Pursey and colleagues [28]. These scoring systems were combined in order to include relevant criteria for both animal [38] and human [28] studies. Criteria included questions about sample selection, study design, and reporting of findings. The authors gave answers of “Yes”, “No”, or “Unclear” regarding each question for every article included in the review. An answer of “non-applicable” was given for the question “Was there sufficient description of the groups?” if a study did not have multiple groups. Quality scores were obtained by summing the number of “Yes”, “No”, and “Unclear” ratings, then calculating the ratio of “Yes” ratings to the sum of the “No” and “Unclear” ratings combined. “Not applicable” ratings were not included in the calculation of the overall quality rating. Disagreements between authors were discussed until a resolution was agreed upon.

## 3. Results

The original database search produced a total of 2421 articles, and the updated search produced 577 articles. Three additional articles describing eight studies were identified from references in other papers. After removing duplicate references and excluding articles that did not meet the predetermined inclusion criteria, a total of 35 articles and 52 studies were identified (see Figure 1 and Appendix, Table A1). Primary reasons for exclusion were study objective (i.e., did not aim to evaluate the validity of the food addiction construct) and study type (i.e., not a quantitative empirical study). Publishing dates of included articles ranged from 1999 to 2017. Twenty articles (comprising 22 studies) involved human participants and 15 articles (comprising 30 studies) involved animal subjects (i.e., rats, mice, and monkeys). Forty-nine studies focused on the addictive potential of certain foods, five studies focused on the addictive potential of eating patterns, and two studies focused on the addictive potential of both certain foods and eating patterns.

As seen in Appendix, Table A2, quality scores for included articles ranged from 0.8 (lowest ratio of “Yes” ratings to “No” ratings plus “Unclear” ratings) [39] to a perfect score (all “Yes” ratings) [40,41,42,43]. Three articles disclosed competing financial interests, including Coca-Cola [44], the International Sweeteners Association [44], sugar industry relations [44], pharmaceutical companies [13,40] and involvement in addiction/impulse disorder organizations [13]. Nineteen articles reported no competing interests and 13 made no statement (see Table A2). As competing financial interests may bias study conclusions [45], data should be objectively considered with this context in mind.

Of the 35 articles (52 studies) included in this review, 31 articles (47 studies) reported results supporting the criteria for addiction, two articles (two studies) were mixed, and two articles (three studies) reported unsupportive findings (see Appendix, Table A3). Results examining support for each pre-specified addiction characteristic were evaluated separately and are described below.

### 3.1. Neurobiological Correlates of Addiction

#### 3.1.1. ∆FosB

Sharma, Fernandes, and Fulton [46] showed that rats placed on a 12-week high-fat diet of primarily hydrogenated coconut oil, maltodextrin, sucrose, and casein had significantly higher ∆FosB, dopamine D2 receptor, and brain-derived neurotropic factor expression, and lower dopamine D1 receptor expression, in the NAc. These changes were observed before the onset of obesity and were linked to behaviors suggestive of anhedonia. The authors concluded that the brain changes may have put the animals at greater risk for addictive-like symptoms such as relapse. No human studies reported findings related to ∆FosB.

#### 3.1.2. Dopamine

Colantuoni and associates [47] compared brain chemistry changes in rats with intermittent, excessive glucose intake to rats given a normal diet of chow. They found that exposure to the highly palatable food in an intermittent eating pattern caused increased activation of dopamine D1 (*p* < 0.05) and μ-opioid-1 receptors (*p* < 0.05), as well as decreased binding of dopamine D2 receptors, in the dorsal striatum (*p* < 0.05). Adams and colleagues [41] found that rats given a high-fat/low-sucrose diet (primarily lard) also had decreased D2 receptor expression in the NAc, but those given a low-fat/high-sucrose diet did not (high fat diet: *F* = 11.1, *p* = 0.009; high sucrose diet *F* = 3.8, *p* = 0.074). Reduced D2 receptor expression (*p* < 0.01) in the striatum (along with other indicators of down-regulation of reward functioning) was also observed in rats who volitionally overate highly palatable foods (bacon, sausage, cheesecake, pound cake, frosting, chocolate) in a study by Johnson and Kenny [48]. Authors of each study concluded that their results were consistent with findings in substance use disorder literature.

In humans, Davis and associates [49] found that individuals who met the YFAS cutoff suggesting clinically significant food addiction symptoms had higher multi-locus genetic profile (MLGP) scores associated with increased dopamine signaling (*p* = 0.023), and that the relationship between the MLGP scores and food addiction was mediated by reward-driven eating (95% CI: 0.00–1.12). Davis, Levitan, Kaplan, Kennedy, and Carter [50] showed that an appetite suppressant that blocked dopamine functioning was not effective in adults who screened positive for food addiction on the YFAS compared with controls, suggesting altered dopamine signaling strength in adults with more food addiction symptoms similar to what is seen among adults with substance use disorders.

#### 3.1.3. Opioid Expression

Opioid receptors were reported to play a role in food reward in rats. Le Merrer and Stephens [51] found that rats conditioned on sugar sweetened pellets no longer responded to the conditioned reward when given an opiate antagonist (naltrexone; dose effect: *F*_2,32_ = 1.72, non-significant). Newman, Pascal, Sadeghian, and Baldo [52] showed that rats who were fed sweetened shortening daily ate significantly more standard chow than rats not fed the palatable food when given a μ-opioid receptor agonist (DAMGO), suggesting that opioid receptor activity may be associated with overeating and consumption of highly palatable foods.

As a measure of opioid function, Daubenmier and colleagues [53] analyzed the effects of an acute opioid blockade drug (naltrexone) on cortisol and nausea in overweight/obese women. They found that women who engaged in more emotional and restrained eating had greater levels of cortisol (*r* = 0.37, *p* < 0.05), and women who engaged in binge eating had greater levels of nausea in response to the drug (*p* = 0.048), suggesting that these individuals “may have a down-regulated opioidergic system” (p. 99). Cambridge and associates [40] similarly found a significant role for the μ-opioid receptor system in motivation for food reward; they observed that a μ-opioid receptor antagonist reduced motivation for, but not liking of, high calorie foods (e.g., chocolate) in people with obesity and moderate binge eating (*p* < 0.05). Although the authors did not conclude that their results supported food as an addictive substance, they did indicate a role for the μ-opioid system in food-related reward.

#### 3.1.4. Other Neurobiological Changes

In a controlled study conducted on primates, Duarte and colleagues [54] found that chocolate induced a persistent conditioned place preference response usually only seen in response to drug rewards. Monkeys who received chocolate spent more time in environments where they had previously received chocolate, whereas controls showed no place preference (*F*_1,13_ = 13.59, *p* = 0.003, *η*^2^*_p_* = 0.53). Conditioning persisted even after a 15-day follow-up (*F*_1,13_ = 4.31, *p* = 0.06, *η*^2^*_p_* = 0.26), indicating that chocolate, like drugs, can be used for this type of conditioning. In rats, Le Merrer and Stephens [51] found that an AMPA receptor antagonist blocked the conditioned response to sweetened pellets (*F*_2,34_ = 3.02, non-significant) in a manner comparable to drugs of abuse. Additionally, Newman and colleagues [52] suggested that gamma-aminobutyric acid (GABA) receptor activity may be implicated in food addiction; they found that rats given a daily dose of sweetened shortening ate significantly more standard chow than rats not fed the palatable food when given muscimol, a GABA agonist that induces feeding (*p*s < 0.01).

In a study comparing rats genetically prone to obesity against rats resistant to obesity, Mary Brown and colleagues [55] reported a significant role for the NAc glutamatergic system in driving overeating (*p*s < 0.05), similar to the glutamatergic mechanisms seen in animal models of relapse to drug addictions. Additionally, Pérez-Ortiz and associates [56] found that rats fed a high-fat diet (primarily lard, casein, and sucrose), exhibited increases in potential biomarkers of addiction (fumarate hydratase, ATP synthase subunit alpha, and transketolase) in the NAc (*p* < 0.05). Adams and colleagues [41], however, found that a high fat diet (primarily lard) reduced activity of cAMP response element-binding protein (CREB; *F*_1,10_ = 5.4, *p* = 0.042) and its activated form (pCREB; *F*_1,10_ = 5.9, *p* = 0.036) in the NAc, contrary to their prediction.

In an electroencephalographic (EEG) study by Imperatori and colleagues [57], participants with three or more food addiction symptoms on the YFAS exhibited brain changes similar to those in people with addictive disorders (e.g., increased functional connectivity in fronto-parietal areas; *p*s < 0.05). A functional magnetic resonance imaging (FMRI) study by Gearhardt and colleagues [12] found that YFAS symptom scores were correlated with increased activation in the amygdala, cingulate cortex, and medial orbitofrontal cortex when participants were anticipating consumption of a chocolate milkshake. When participants received the milkshake, those with higher YFAS scores had greater activation in the dorsolateral prefrontal cortex (*p* = 0.007) and caudate (*p* = 0.004) and less activation in the lateral orbitofrontal cortex (*p* = 0.009) compared to those with lower YFAS scores. Gearhardt and colleagues concluded that this pattern of increased activation in areas of the brain related to reward and decreased activation in areas related to inhibition is similar to that seen in substance dependence.

De Ridder and colleagues [58] compared resting-state EEG brain activity between (1) adults with obesity who endorsed more than three YFAS symptoms (“High YFAS”), (2) adults with obesity who endorsed less than three YFAS symptoms (“Low YFAS”), (3) adults without obesity or food addiction (“Lean controls”), and (4) adults without obesity but with alcohol use disorder (“Alcohol addiction”). Positive correlations were found between YFAS symptoms and the rostral anterior cingulate cortex (rACC) for theta (*r* = 0.23, *p* = 0.041) and beta3 (*r* = 0.22, *p* = 0.041) frequency bands. Increased gamma activity in the rostral anterior cingulate cortex (rACC) extending to the dorsal medial prefrontal cortex (dmPFC) was associated with increased hunger ratings in the High YFAS group only (*r* = 0.72, *p* = 0.002), and increased alcohol craving in the Alcohol addiction group (*r* = 0.72, *p* = 0.002), while the rACC was negatively correlated with hunger in the Low YFAS group. Conjunction analyses further revealed similarities between the High YFAS group and the Alcohol addiction group in the ACC/dmPFC and precuneus (*Z* = 2.24, *p* = 0.013), sgACC, orbitofrontal cortex (OFC), and temporal lobe (fusiform/parahippocampal area) (*Z* = 2.78, *p* = 0.003). No correlations were found between the Low YFAS and Alcohol addiction groups. The authors concluded that there were significant neurobiological similarities between persons with food addiction symptoms and alcohol dependence.

In a gustatory cue exposure trial among overweight/obese adolescents, Feldstein Ewing and associates [59] found that consumption of high-calorie beverages (Sprite, Fanta, or Coca Cola) produced brain responses (e.g., increased activation in the nucleus accumbens, cerebellum, bilateral OFC, etc.) similar to those observed in response to addictive drugs. Unlike Gearhardt and colleagues, however, they found no significant relationship between YFAS symptoms and brain response.

Finally, Franken and colleagues [60] found that, similar to patterns seen in drug addiction, individuals with more YFAS symptoms displayed more impairments in cognitive control and performance monitoring on behavioral (Flanker task; *r* = 0.39, *p* = 0.001) and neurological (EEG; *p* < 0.05) tests.

### 3.2. Preoccupation with Substance Use

Given obvious constraints, no animal studies assessed the characteristic of “preoccupation” as it relates to food addiction. However, Tuomisto and colleagues [61] found that self-identified chocolate addicts were significantly more susceptible to hunger compared to controls, possibly indicating a heightened preoccupation with food (*F*_1,26_ = 11.65, *p* < 0.005). Additionally, Merlo and colleagues [18] found that in children, food addiction symptoms (measured by the Eating Behaviors Questionnaire) were significantly associated with greater preoccupation with food (*r* = 0.58, *p* < 0.001; measured by the Children’s Eating Attitude Test).

### 3.3. Impaired Control

#### 3.3.1. Substance Used in Larger Amounts or over a Longer Period than Intended

Burmeister, Hinman, Koball, and Hoffmann [62] found that in a sample of treatment-seeking adults with obesity, the number of addictive-like eating symptoms endorsed on the YFAS was associated with greater self-reported difficulty controlling eating in certain situations (e.g., when nervous or in social settings; *r* = 0.59, *p* < 0.01). In their pediatric sample, Merlo and colleagues [18] found a significant positive association between the uncontrolled eating subscale on the Three Factor Eating Questionnaire and symptoms of food addiction (*r* = 0.60, *p* < 0.001) on the Eating Behaviors Questionnaire.

#### 3.3.2. Persistent Desire or Unsuccessful Efforts to Cut Down or Control Substance Use

No studies specifically evaluated this sub-criterion.

#### 3.3.3. Great Deal of Time Spent Obtaining, Using, or Recovering from the Effects of the Substance

Furlong and colleagues [42] randomly assigned rats to either restricted, continuous, or no access to sweetened condensed milk for five weeks. They reported that, compared to the continuous access and no access (control) conditions, rats given restricted access to sweetened condensed milk exhibited more habitual behavior and time attempting to obtain the treat (i.e., continuing to press a lever, despite the absence of its conditioned reward). Mary Brown and colleagues [55] reported that rats who became obese due to overconsumption of highly palatable food (consisting mostly of lard, casein, and sucrose) also exhibited more perseverative behaviors related to highly palatable foods by lever pressing in the absence of a reward (*t* = 3.76, *p* = 0.006), greater motivation for the palatable food (*t =* 3.755, *p* = 0.006), and greater number of lever presses (*t* = 2.87, *p* = 0.007) compared to rats on the same diet who did not become obese. 

#### 3.3.4. Craving, or a Strong Urge to Use the Substance

Lenoir and associates [43] found that rats preferred saccharin over cocaine when given the choice (*p*s < 0.05), and were more willing to work for saccharin in the face of increased cost (*p*s < 0.05), indicating a strong desire to consume the sweetener.

Davis and colleagues [39] found that adults with obesity who met YFAS criteria for food addiction reported significantly greater food cravings (*p* < 0.001), hedonic eating (*p* < 0.001), and snacking on sweets (*p* < 0.001). In a separate study, Davis and associates [49] reported that participants with clinically significant food addiction symptoms (based on the YFAS) had higher scores on a measure of food cravings (*F* = 55.07, *p* < 0.001) compared to those without, and found that craving mediated the relationship between dopamine signaling and clinically significant food addiction symptoms (95% CI: 0.04–0.93), even after controlling for binge eating and emotional eating. Additionally, Davis and colleagues [50] demonstrated that individuals who met the YFAS criteria for clinically significant food addiction symptoms reported greater food cravings and appetite following a taste of their favorite palatable food (e.g., potato chips, chocolate, cookies; *p* < 0.001).

In a study of bariatric surgery candidates with binge eating disorder, Lent and Swencionis [63] found that food cravings were associated with higher scores on a measure of addictive personality (*r* = 0.31, *p* = 0.005) and that addictive personality scores explained a significant amount of the variance in cravings (*R*^2^ = 0.10, *p* = 0.005). Tuomisto and colleagues [61] found that self-identified “chocolate addicts” were more subjectively aroused and reported experiencing greater cravings when presented with chocolate related cues (e.g., sight, smell, taste) than controls (*p* < 0.001). Finally, Feldstein Ewing and associates [59] observed significant increases in adolescent boys’ self-reported urges to eat after tasting a sweetened beverage (e.g., Sprite, Fanta, or Coca Cola) compared to water (*t* (23) = 2.20, *p* = 0.04).

### 3.4. Social Impairment

#### 3.4.1. Failure to Fulfill Major Role Obligations at Work, School, or Home due to Recurrent Substance Use

No studies specifically evaluated this sub-criterion.

#### 3.4.2. Continued Use Despite Social or Interpersonal Problems Caused/Exacerbated by Substance 

Adams and colleagues [41] found that rats fed a calorie-restricted high-fat/low-sucrose diet began seeking sucrose rewards more impulsively, even when impulsive behavior was punished with time-out from other rats (*F*_1,11_ = 6.4, *p* = 0.028). However, rats fed the low-fat/high-sucrose diet did not show the same level of impulsive behavior (*F*_1,12_ = 1.2, *p* = 0.297). No human studies specifically evaluated this sub-criterion.

#### 3.4.3. Reduction of Important Social, Occupational, or Recreational Activities due to Substance Use

No animal studies evaluated this sub-criterion; however, Lent and Swencionis [63] found that 60% of their sample of bariatric surgery candidates endorsed choosing to spend time eating over conducting other activities, and that this subgroup also had higher addictive personality scores (*p* < 0.01). In turn, higher scores on their addictive personality measure explained a significant amount of the variance in social isolation (*R*^2^ = 0.28, *p* < 0.001).

### 3.5. Risky Substance Use

#### 3.5.1. Recurrent Substance Use in Physically Hazardous Situations

Johnson and Kenny [48] observed that rats given unrestricted access to a diet consisting of bacon, sausage, cheesecake, pound cake, frosting, and chocolate continued to compulsively consume these foods despite the presence of an aversive conditioned stimulus (i.e., a cue light that had previously been paired with foot shock; *F*_1,26_ = 29.7, *p* < 0.001). In contrast, rats previously fed only regular chow and/or given restricted access to the high-fat/high-sugar diet significantly decreased their palatable food consumption in the presence of the aversive conditioned stimulus (*F*_1,26_ = 44.9, *p* < 0.001). No human studies evaluated this sub-criterion.

#### 3.5.2. Continued Use Despite Knowledge of Physical or Psychological Problem Likely Caused or Exacerbated by the Substance

No studies specifically evaluated this sub-criterion.

### 3.6. Pharmacological Criteria

#### 3.6.1. Tolerance

Johnson and Kenny [48] found that rats who volitionally overate highly palatable food exhibited reward dysfunction (e.g., downregulated dopamine D2 receptors, elevated reward thresholds) that worsened as the rats gained more weight (*F*_2,6_ = 5.2, *p* < 0.05).

Among bariatric surgery candidates in Lent and Swencionis’s study [63], 68.5% reported increasing quantities of food to reach satiation, and those who endorsed this symptom also had higher scores on the addictive personality measure. Additionally, Spring and associates [64] showed that among women who reported craving carbohydrates, a 100% carbohydrate sweetened beverage (including sucralose, maltodextrin, dextrose, high maltose rice syrup, etc.) significantly dispelled negative mood (*t* (789) = 2.17, *p* = 0.03). However, this effect decreased over multiple exposures, indicating signs of tolerance among this sample (*t* (95) = −2.82, *p* = 0.01). Finally, Markus and colleagues [44] reported that the most common foods associated with tolerance-like effects in their study were high-fat sweet foods (3.2%) and high-fat savory foods (2.9%), and that the “intensity” of tolerance was greater for these foods compared to low-fat sugary foods (*p*s < 0.05). The authors did not report how “intensity” was measured.

#### 3.6.2. Withdrawal

Mangabeira, Garcia-Mijares, and Silva [65] found that rats who preferred a sugar solution had impaired differential reinforcement of low rate performance (a measure of impulsivity) when forced into abstinence (*p* < 0.001), similar to animals in withdrawal from addictive drugs. Pickering, Alsiö, Hulting, and Schiöth [66] found that when given a high-fat/high-sugar diet, obesity-prone rats exhibited withdrawal symptoms, including spending less time in the center of an open-field test (an indicator of anxiety; *p* < 0.05) and eating significantly less regular chow (*p* < 0.05) upon removal of the high-fat/high-sugar diet. Sharma and colleagues [46] reported that upon withdrawal from their diet, mice fed high-fat food (primarily hydrogenated coconut oil, maltodextrin, sucrose, and casein) showed more signs of anxiety and increased basal cortisone levels (*p* < 0.01), and the rats were more motivated for both sucrose and high-fat foods compared to rats fed a low-fat diet (*p* < 0.01). However, Yakovenko, Speidel, Chapman, and Dess [67] reported that spontaneous withdrawal symptoms of rats reported in other studies (forepaw tremor, teeth chatter, and head shake) were rare in their study. While they did observe elevated startle (a symptom of ethanol withdrawal seen in the same line of rats) dependent upon the prior dose of glucose intake (*r* = 0.63), it was not significantly different from controls (*p* > 0.10).

Lent and Swencionis [63] found that, in their sample of bariatric surgery candidates, individuals who scored significantly higher on an addictive personality measure also reported feeling more anxious when they were not near food (*p* < 0.01). Finally, Markus and associates [44] reported that among 1414 participants who reported experiencing at least one YFAS symptom in the past year, 9.5% endorsed “withdrawal-like” physiological effects in response to either high-fat savory foods (3.8%), high-fat sweet foods (2.8%), low-fat sugary foods (1.6%), or low-fat savory foods (1.3%). The self-reported “intensity” of withdrawal symptoms were significantly greater for high-fat savory and high-fat sweet foods compared to low-fat sugary foods (*p*s < 0.05).

### 3.7. Chronicity

McGee, Amare, Bennett, and Duncan-Vaidya [68] found that three days of a binge/compensate pattern of eating sweetened vegetable shortening still had a significant impact on rats’ motivation for sucrose over one month later (*F*_2,19_ = 7.72, *p* < 0.01), suggesting long-term effects of palatable food consumption. Pickering and colleagues [66] reported that obesity-prone rats fed a high-fat/high-sugar diet consumed significantly less chow during a three-week withdrawal period from those foods (*p* < 0.05), possibly suggesting long-term changes to the rats’ reward system analogous to the chronic state of dependence seen in drug addictions.

Konkolÿ Thege, Woodin, Hodgins, and Williams [5] described a longitudinal study in which they evaluated the prevalence of six potentially-addictive behaviors among 4121 Canadian adults. They found that only 6.3% of participants reported problems with excessive overeating for four consecutive years compared to 58% reporting problems for one year only. The authors concluded that excessive overeating may be more transient than drug addictions.

### 3.8. Relapse

Two articles by Pickering and colleagues [66] and Sharma and colleagues [46] reported that rats and mice withdrawn from a highly palatable food diet demonstrated increased motivation for sucrose, suggesting risk for relapse (*F*_1,390_ = 4.71, *p* = 0.0491 and *p*s < 0.01, respectively). No human studies evaluated this criterion.

### 3.9. Additional Observations

#### 3.9.1. Genetics

In a genome-wide investigation of food addiction, Cornelis and associates [69] observed that food addiction scores on the modified YFAS were significantly associated with signaling in the mitogen-activated protein kinase pathway, which has been identified as a possible drug addiction pathway (*p* = 0.02); however, other addiction-related genetic underpinnings did not overlap (e.g., genes, single-nucleotide polymorphisms) with food addiction (*p*s > 0.05).

#### 3.9.2. Substance Sensitization

Le Merrer and Stephens [51] observed that mice exposed to sweetened pellets paired with a specific context displayed signs of behavioral sensitization by showing greater progressive activity in that context compared to mice that did not have the same pairing (*p* < 0.05). This activity persisted for three weeks in the absence of the palatable food and was described as being similar to those seen in models of drugs sensitization. These authors also reported that conditioned environments produced greater food consumption. 

In humans, Spring and colleagues [64] observed that participants who reported craving carbohydrates endorsed increased “liking” for a pure carbohydrate beverage over time, compared to a control high-protein beverage (*t* (98) = 1.98, *p* = 0.05), which the authors concluded suggested sensitization to carbohydrates in this sample.

#### 3.9.3. Cross-Sensitization

Le Merrer and Stephens [51] reported that rats sensitized to palatable food had a significantly enhanced locomotor response when given cocaine and morphine (*F*_1,18_ = 6.18, *p* < 0.05), suggesting a cross-sensitization effect. However, Yakovenko and colleagues [67] failed to find evidence for a cross-sensitization effect of cookie consumption on alcohol intake among rats. 

In a study on weight-loss surgery patients, Fowler, Ivezaj, and Saules [70] observed that those who reported more problems with high glycemic index and high-sugar/low-fat foods before surgery were more likely to develop a new substance use disorder post-surgery (*p*s < 0.05), indicating cross-sensitization.

#### 3.9.4. Impulsivity

Adams and colleagues [41] found that rats fed a high-fat diet showed increased impulsivity in working for a sucrose reward compared to rats fed a high-sugar diet (*F*_1,11_ = 6.4, *p* = 0.02). In humans, Davis and associates [39] reported greater impulsivity among adults with obesity who met YFAS criteria for food addiction compared to controls who did not (*p* < 0.001).

### 3.10. Overall Addiction

Four studies reported results relevant to an overall characterization of addiction. Tuomisto and associates [61] compared self-identified “chocolate addicts” to “non-chocolate addicts” in two studies and found that exposure to chocolate cues led to affective changes (e.g., anxiety, restlessness) similar to those seen in substance addiction. Additionally, Schulte, Avena, and Gearhardt [13] evaluated whether certain foods were more likely to be associated with addictive-like eating in undergraduates and in a more diverse sample of adults recruited through Amazon MTurk, respectively. Participants in their study completed the YFAS and then were asked to complete a forced-choice task of identifying which foods were associated with addictive symptoms. The authors reported that the foods most likely to be implicated in addictive-like eating patterns were processed foods high in fat and/or refined carbohydrates. These foods, the authors stated, parallel pharmacokinetic properties of addictive drugs (e.g., highly concentrated, rapid absorption rate).

## 4. Discussion

The concept of food addiction has sparked much controversy among researchers. While some have questioned the validity of this construct [25,30], an increasing number of studies have produced evidence of biological and behavioral changes in response to highly palatable foods that parallel addiction criteria. The current study reviewed existing food addiction research and organized the findings into key diagnostic constructs: (a) neurobiological changes, (b) preoccupation with the substance, (c) impaired control, (d) social impairments, (e) risky use, (f) tolerance/withdrawal, (g) chronicity of the condition, and (h) relapse. We found significant support for the construct of food addiction in both animals and humans, with each primary criterion having support from at least one study (see Appendix, Table A3), though some sub-criteria have not yet been studied. Of the addiction characteristics assessed in this review, brain reward changes and impaired control had the greatest number of supportive findings (21 and 12 studies, respectively). The current review also found evidence for supplemental characteristics consistent with addiction, including genetic susceptibility, substance sensitization and cross-sensitization, and impulsivity. More research is needed to evaluate the diagnostic criteria with less empirical support, including risky use, chronicity, relapse, preoccupation, and social impairment. Only four of the 35 eligible articles reported findings contrary to the proposed criteria for addiction [5,41,59,67], yet two of these also included supportive findings [41,59]. Overall, evidence supporting the validity of food addiction significantly outweighed evidence against it.

Some have proposed that food addiction should be classified as a behavioral disorder (i.e., “eating addiction”) similar to a gambling disorder [71]. However, most research studies, including the vast majority of studies identified for this review, have conceptualized food addiction as a substance use disorder (i.e., “refined food use disorder”, “highly palatable food use disorder”, or simply “food use disorder” [14,19,72,73,74,75]. To address this question, Meule and Gearhart [14] compared diagnostic criteria for gambling disorder—the only officially recognized *behavioral* addiction in the DSM-5—to symptoms of food addiction and found that despite several similarities (e.g., unsuccessful efforts to cut down), food addiction symptoms more closely resembled those of a substance use disorder due to the necessary consumption of a *substance* (food) and the inapplicability of certain behavioral criteria (e.g., monetary loss: DSM-5 criteria 1, 6, and 5).

By definition, behavioral addictions involve dependence on a behavior, not a substance; however, addictive-like consumption of highly palatable food involves both a behavior (eating) and substance (food). Some classic substance addictions, such as tobacco use disorder, also appear to include behavioral dependencies. For example, behavior modification is typically required in treatment for tobacco use disorder due to the strong connection between the effects of the substance (tobacco) and the act of using it (e.g., smoking) [76]. Nevertheless, tobacco is the primary driver of the addiction, and it is therefore classified as a substance use disorder. In the current review, symptoms suggestive of “addiction” to highly palatable foods were often intertwined with specific eating patterns (i.e., restriction, binge eating) [42,52,67]. However, these behavioral patterns are also frequently observed among individuals with alcohol and other drug use disorders. In addition, characteristics of food addiction were found in the absence of such eating patterns (e.g., [48]) and were preceded by consumption of highly palatable foods, suggesting a profile most similar to substance addiction. In light of these findings, the results of the current review support Meule and Gearhardt’s [14] conclusion that, while food addiction involves both behavioral and substance-related symptoms, it more closely parallels criteria for substance use disorder.

Overall, the majority of the studies in the present systematic review evaluated foods with added sweeteners (e.g., sugar, saccharine), and many experiments combined sweeteners with fats such as hydrogenated oils or lard (see Appendix, Table A1). The current review found that the most common foods associated with addictive symptoms were those high in added fats and/or refined carbohydrates such as sugar. These findings are consistent with prior literature. Avena, Rada, and Hoebel [77] found that neural adaptations in response to sugar consumption could lead to dependence in rats, and Ifland and colleagues [19] concluded that refined foods (e.g., sodas, breakfast cereal, high-fructose corn syrup) should be considered a “classic” addictive substance. Taking it one step further, Lustig and colleagues [73] argued that sugar should be regulated as substance of abuse given the negative health outcomes common to both sugar and alcohol at the individual and societal levels (e.g., liver disease, associated medical costs). Schulte, Potenza, and Gearhardt [75] proposed that food addiction more closely resembles a substance-based addiction as opposed to a behavioral addiction due to the differential effects of certain foods types on eating behavior. Finally, Pursey and colleagues [34] reviewed the literature on food addiction and concluded that the foods most commonly associated with addictive-like symptoms in humans are those that are highly-processed, high on the glycemic index, and contain large amounts of added fats and sugar. Although there is strong support for the addictive potential of sugar in animal studies [77], data from human studies suggest that the *combination* of sweet and fat is more commonly associated with addictive symptoms than sugar alone [34,44]. More research is needed to identify the types and characteristics of food ingredients that may have addictive effects in humans.

Few studies have evaluated whether food addiction can manifest in response to consumption of unprocessed “whole foods.” Animal studies in the current review found no evidence for addictive-like symptoms to rodent chow (e.g., [48]), and human studies reported increased addictive symptoms toward refined/processed foods compared to non-processed foods [13]. Nevertheless, in a study by Schulte and colleagues [13] evaluating the addictive potential of specific foods, nuts (typically considered a whole food, without added sugars) were rated more addictive on average than granola bars (typically processed, with added sugars and fats). Furthermore, there was an isolated report describing individuals who displayed addictive-like symptoms toward carrots [78]. While highly palatable foods are associated with more addictive-like symptoms than non-processed foods, the possibility of these symptoms occurring in response to “natural” food merits further exploration.

Future research should also examine potential biological and hormonal factors that play a role in food addiction symptoms. Studies in this review found that rodents genetically prone to obesity had greater risk for developing certain food addiction symptoms (i.e., craving, impaired control) compared to obesity-resistant rodents [48,66]. In humans, symptoms of food addiction are more prevalent among adults in the overweight and obese BMI categories (24.9%) compared to adults in the normal BMI category (11.1%) [28]. However, a study comparing adults with overweight/obesity, found hormonal differences (e.g., amylin, prolactin, thyroid stimulating hormone) between those who met criteria for food addiction and those who did not [79]. These data indicate a need to further explore the biological and hormonal factors associated with both weight and food addiction.

Finally, while multiple studies have shown that obesity, binge eating disorder, and food addiction are separate constructs [26,27], their distinct etiologies leave much to be clarified. Future research should continue to examine the neurological correlates and differences between obesity, eating disorders, and food addiction. Potential theoretical and clinical implications of these differences should be explored.

To our knowledge, this is the first systematic review on food addiction that was not limited to definitions based on the YFAS or body weight status. Strengths include the use of human and animal studies, rigorous methodology using PRISMA guidelines and inclusion of both animal and human studies. Limitations include that our search was limited to two electronic databases and only included studies published in English, and that animal studies limit generalizability to humans. In addition, our risk of bias assessment may have resulted in lower scores for older studies, due to changes over time in reporting standards (e.g., financial support, conflicts of interest). The study question may also have produced biased results, as researchers interested in evaluating the validity of the food addiction construct may be more inclined (consciously or not) to observe and report confirmatory results. When combined with publication bias, this may have resulted in an underrepresentation of studies producing contrary or null findings. Finally, our search criteria likely excluded evidence for certain characteristics of addiction (e.g., social impairment, risky use, preoccupation) because these constructs are relatively difficult to measure quantitatively. However, these symptoms have been reported in qualitative studies (e.g., [80]) and are plausible when considering, for example, individuals who continue to overeat post-bariatric surgery or despite exacerbated chronic medical conditions such as diabetes or heart disease [14,26]. As recommended by Burrows and colleagues [26], future reviews on food addiction could benefit from including both quantitative and qualitative studies.

## 5. Conclusions

The results of the current systematic review generally support the validity of food addiction as a diagnostic construct, particularly as it relates to foods high in added sweeteners and refined ingredients. The majority of studies in the current review reported evidence for symptoms related to neurological changes and impaired control, with fewer studies evaluating preoccupation, chronicity, relapse, social impairment, and risky use. Behavioral and substance-related aspects of food addiction appear to be intertwined, but we suggest that the substance (highly-palatable food) component may be more salient to the diagnostic classification of this phenomenon than the behavior (eating). We propose that the food addiction construct merits serious attention in regard to its presentation, prevention, and treatment in humans.

## Figures and Tables

**Table 1:** Definitions of addiction.

Source	Definition
English Oxford Dictionary [15]	“Physically and mentally dependent on a particular substance.”
American Psychological Association [16]	“A chronic disorder with biological, psychological, social and environmental factors influencing its development and maintenance. Genes affect the degree of reward that individuals experience when initially using a substance (e.g., drugs) or engaging in certain behaviors (e.g., gambling), as well as the way the body processes alcohol or other drugs. Heightened desire to re-experience use of the substance or behavior, potentially influenced by psychological (e.g., stress, history of trauma), social (e.g., family or friends’ use of a substance), and environmental factors (e.g., accessibility of a substance, low cost) can lead to regular use/exposure, with chronic use/exposure leading to brain changes.”
American Society of Addiction Medicine [10]	“A primary, chronic disease of brain reward, motivation, memory and related circuitry. Dysfunction in these circuits leads to characteristic biological, psychological, social and spiritual manifestations. This is reflected in an individual pathologically pursuing reward and/or relief by substance use and other behaviors. Addiction is characterized by inability to consistently abstain, impairment in behavioral control, craving, diminished recognition of significant problems with one’s behaviors and interpersonal relationships, and a dysfunctional emotional response. Like other chronic diseases, addiction often involves cycles of relapse and remission. Without treatment or engagement in recovery activities, addiction is progressive and can result in disability or premature death.”
American Psychiatric Association [17]	“A complex condition, a brain disease that is manifested by compulsive substance use despite harmful consequence. People with addiction (severe substance use disorder) have an intense focus on using a certain substance(s), such as alcohol or drugs, to the point that it takes over their life. They keep using alcohol or a drug even when they know it will causes problems. Yet a number of effective treatments are available and people can recover from addiction and lead normal, productive lives.”

**Table 2:** Substance Use Disorder criteria, adapted from the Diagnostic and Statistical Manual of Mental Disorders, 5th Edition (DSM-5) [1].

**Impaired Control** Consuming a substance in greater amounts or over longer periods of time than intended. Having a persistent desire or unsuccessfully attempting to decrease or limit substance use. Spending a significant amount of time acquiring, using, or recovering from a substance. Craving the substance or having a strong urge to use it.
**Social Impairment** 5. Being unable to fulfill obligations at work, school, or home due to use of a substance. 6. Continually using a substance despite its effects causing or exacerbating persistent or recurrent social or interpersonal problems. 7. Giving up or reducing social, occupational, or recreational activities due to substance use.
**Risky Use** 8. Continually using a substance in situations in which it is physically dangerous (e.g., driving under the influence of a substance). 9. Continually using a substance despite physical or psychological problems that are caused or made worse by the substance use.
**Pharmacological Criteria** 10. Needing a substantially higher dose of the substance to achieve the desired effect; or experiencing a substantially reduced effect of the substance when the usual dose is consumed (i.e., tolerance). 11. Experiencing negative physical and psychological symptoms when the substance is not consumed at the typical dose or frequency (i.e., withdrawal).

*Note.* To meet DSM-5 criteria for a substance use disorder, clinical distress or impairment must be evidenced by two or more of the above symptoms within a 12-month period. Severity is classified as mild (2–3 symptoms), moderate (4–5 symptoms), or severe (≥6 symptoms).

## Appendix A

**Table A1:** Characteristics of included studies.

First Author (Year)	Study Type	Sample Characteristics	*n* (Experimental/Control)	Independent Variable(s)	Outcome(s)
Adams (2015) [41]	Animal	Male Long-Evans rats	16/8	Restricted, equicaloric high-fat/low-sugar vs. low-fat/high-sugar diets	Impulsivity and attention, measured by the five-choice serial reaction time task; dopamine signaling in the dorsal and ventral striatum; insulin and leptin levels in the plasma
Burmeister (2013) [62]	Cross-sectional	Adults with obesity in a behavioral weight-loss program Female: 68.4% Age: 47.4 years BMI: 38.2 kg/m^2^ Caucasian: 84.2%	57/0	Food addiction symptoms (YFAS continuous)	7-week weight change; measures of psychological distress, disordered eating, weight bias, and weight-focused attitudes
Cambridge (2013) [40]	Double-blind, placebo-controlled parallel group study	Adults with obesity and moderate binge eating Female: 53.3% Age: 40.2 years	16/14	Mu-opioid receptor antagonist (GSK1521498) or placebo	Brain responses to food images (FMRI and behavioral measures); motivation to expend energy to view comparable images
Colantuoni (2001) [47]	Animal	Female Sprague-Dawley rats	10/5	“Intermittent excessive sugar intake” (25% glucose solution with chow for 12 h followed by 12 h of food deprivation each day)	Receptor binding (e.g., dopamine, opioid)
Cornelis (2016) [69]	Genome-wide association study	Women of European ancestry participating in the Nurses’ Health Study Age: 25–55 years at start	9314/0	Food addiction (mYFAS)	Enrichment of SNPs, genes, and pathways implicated in drug addiction
Daubenmier (2014) [53]	Cross-sectional	Women in a waitlist control for a randomized controlled trial of a mindfulness intervention for stress eating Age: 40.9 years BMI: 31.1 kg/m^2^	16/17	Naltrexone-induced nausea and cortisol levels (measure of central opioidergic activity)	Indices of hedonic-related eating behaviors (binge, emotional, external, or restrained eating); intake of sweets/desserts, carbohydrates; interoceptive awareness; adiposity; weight change
Davis (2011) [39]	Cross-sectional	Adults with obesity Female: 68.1% Age: 33.6 years BMI: 38.5 kg/m^2^ Caucasian: 81.4%	18/54	Food addiction (YFAS dichotomous)	Clinical co-morbidities (e.g., binge eating disorder, attention deficit hyperactivity disorder), psychological risk factors (e.g., impulsivity), and abnormal motivation for the addictive substance
Davis (2013) [49]	Case-control	Adults recruited for study on overeating/overweight Female: 68.3% Age: 25–47 years	21/99	Composite index of elevated dopamine signaling (a multi-locus genetic profile score)	Food addiction (YFAS dichotomous); eating-related sub-phenotypes of food addiction (e.g., binging)
Davis (2014) [50]	Three-way mixed model, double-blind cross-over	Adults, predominately overweight/obese Female: 67.7% Age: 32.7 years BMI: 33.9 kg/m^2^	23/113	Food addiction (YFAS dichotomous); psychomotor stimulant (methylphenidate) vs. placebo	Appetite, cravings, and consumption of favorite snack
De Ridder (2016) [58]	Cross-sectional	Adults Female: 79.3% Age: 45.1 years BMI: 33.2 kg/m^2^	38/34	Weight category (normal vs. obese BMI); food addiction (YFAS continuous)	EEG; hunger; behavioral inhibition; eating style; binge eating; food awareness
Duarte (2014) [54]	Animal	Marmoset monkeys Female: 50%	6/8	15 min exposure to 50 g chocolate	Conditioned-place-preference
Feldstein Ewing (2017) [59]	Cross-sectional	Youth, overweight/obese Male: 83.3% Age: 16.5 years BMI: 33.1 kg/m^2^ Hispanic: 79%	24/0	Beverage type (sweetened soft drink vs. water); food addiction (YFAS continuous); BMI; insulin resistance	Urge to eat; FMRI response patterns (BOLD activation)
Fowler (2014) [70]	Secondary data analyses	Adults, 2.7 years post-bariatric surgery Female: 88.4% Age: 48.7 years BMI: 32.3 kg/m^2^ Caucasian: 94.2%	154/0	Pre-surgical problems with high-sugar/low-fat foods and foods with a high glycemic index	Risk for new onset substance use disorder post-surgery
Franken (2016) [60]	Cross-sectional	Students recruited for larger YFAS study Age: 20.4 years BMI: 21.7 kg/m^2^	34/34	Food addiction (YFAS continuous)	Cognitive control (error monitoring) via Eriksen flanker task and EEG (ERN, Pe)
Furlong (2014) [42] study 1	Animal	Male Long-Evans rats	24/12	Continuous vs. restricted access to sweetened condensed milk (3:1 ratio of Nestle to water)	Goal-directed performance and neuronal activity in corticostriatal circuits
Furlong (2014) [42] study 2	Animal	Male Long-Evans rats	8/10	AMPA-receptor and dopamine D1-receptor antagonists	Habitual performance following restricted access to a highly palatable food
Gearhardt (2011) [12]	Cross-sectional	Young women Age: 20.8 years BMI: 28.0 kg/m^2^	39/0	Food addiction symptoms (YFAS continuous)	FMRI patterns of neural activation similar to substance dependence (in response to actual and anticipated receipt of chocolate milkshake)
Imperatori (2015) [57]	Cross-sectional	Adults with overweight or obesity admitted to a medical center for obesity treatment Female: 78.6% Age: 43.6 years BMI: 28.5 kg/m^2^	14/14	Food addiction symptoms (I-YFAS continuous and dichotomous); taste of chocolate milkshake	EEG modifications and connectivity
Johnson (2010) [48] study 1	Animal	Male Wistar rats	22/9	Restricted vs. extended access to “cafeteria style” diet (e.g., bacon, sausage, cheesecake, frosting)	Brain stimulation reward threshold; body weight change; caloric intake; type of food consumed (cafeteria vs. chow)
Johnson (2010) [48] study 2	Animal	Male Wistar rats	Not reported	Restricted vs. extended access to cafeteria style diet; body weight; knockdown of striatal dopamine D2 receptor	Reward hyposensitivity (measured by striatal D2 receptor density); brain stimulation reward threshold; body weight change; caloric intake; type of food consumed (cafeteria vs. chow)
Johnson (2010) [48] study 3	Animal	Male Wistar rats	Not reported	Restricted vs. extended access to cafeteria style diet followed by intermittent access (30 min) to cafeteria food; environmental stimulus (light) predicting adversity (foot shock)	Brain stimulation reward threshold; body weight change; caloric intake; type of food consumed (cafeteria vs. chow); compulsive-like eating behavior
Johnson (2010) [48] study 4	Animal	Male Wistar rats	Not reported	Restricted vs. extended access to cafeteria style diet; knockdown of striatal dopamine D2 receptor; environmental stimulus (light) predicting foot shock	Brain stimulation reward threshold; caloric intake; type of food consumed (cafeteria vs. chow); compulsive-like eating behavior
Konkolÿ Thege (2015) [5]	Longitudinal study	Adults Female: 54.7% Age: 46.1 years	4121/0	Over-involvement (causing significant problems) in one of six excessive behaviors (including eating)	Prevalence, substance use comorbidity, five-year trajectory
Le Merrer (2006) [51] studies 1 & 2	Animal	Male mice	20/10	Sweetened pellets while hungry vs. while satiated	Behavioral sensitization (assessed by locomotor activity in sweetened-pellet-paired environment)
Le Merrer (2006) [51] study 3	Animal	Male mice	9-10/0	Dopaminergic agonists (SCH23390, sulpiride)	Pellet-induced conditioned activity
Le Merrer (2006) [51] study 4	Animal	Male mice	20/0	Opiate (naltrexone) and AMPA (GYKI 52466) receptor antagonists	Pellet-induced conditioned activity
Le Merrer (2006) [51] study 5	Animal	Male mice	20/0	Cocaine or morphine; pretreatment of GYKI 52466, naltrexone, or SCH23390	Cross-sensitization (pellet-induced conditioned activity following cocaine or morphine injection)
Le Merrer (2006) [51] study 6	Animal	Male mice	7–9/0	Sweetened-pellet-conditioned environment	Consumption of sweetened pellets; locomotor activity
Lenoir (2007) [43]	Animal	Young male Wistar rats	132/0	Mutually exclusive choice between sweetened water and intravenous cocaine; history of cocaine preference	Preferred substance (saccharin, sugar, or cocaine)
Lent (2012) [63]	Cross-sectional	Adult bariatric surgery candidates Female: 85.6% Age: 41.0 years BMI: 45.2 kg/m^2^ Caucasian: 67.0%	97/0	Addictive personality (Eysenck Personality Questionnaire Addiction Scale)	Maladaptive eating behaviors (Overeating Questionnaire; binge-eating questions from Questionnaire of Eating and Weight Patterns; Eating Attitudes and Behaviors Questionnaire)
Mangabeira (2015) [65]	Animal	Male Wistar rats	14/14	Withdrawal from prolonged sugar consumption	Impulsivity (assessed by differential reinforcement of low rate performance)
Markus (2017) [44]	Cross-Sectional	Undergraduates Female: 69.9% Age: 21.6 years	1495/0	Food addiction (YFAS dichotomous, continuous}	Depressive symptoms; BMI; YFAS; “problem foods” (high-fat savory, high-fat sweet, low-fat sugary, low-fat savory)
Mary Brown (2015) [55]	Animal	Male Sprague-Dawley rats	Not reported	Propensity to diet-induced obesity	Addictive-like behavior (i.e., heightened motivation; excessive intake; increased food seeking); synaptic impairments in NAc
McGee (2010) [68]	Animal	Male Long–Evans rats	16/8	Withdrawal from intermittent access to a sweet fat mixture	Motivation (operant performance for sucrose on progressive ratio schedule); craving (lever pressing for palatable food); anxiety (elevated plus maze)
Merlo (2009) [18]	Cross-sectional	*Children* Female: 64% Age: 13.8 years BMI: 35.6 kg/m^2^ Caucasian: 60% *Parent/guardian* Female: 87% Age: 43.2 years BMI: 33.0 kg/m^2^	50/0 children and their parent/guardian	BMI; Food addiction symptoms (Eating Behaviors Questionnaire)	Food- and eating-related attitudes and behaviors
Newman (2013) [52]	Animal	Male Sprague-Dawley rats	10/11	Bouts of sweetened-fat intake (shortening with 10% sucrose); predator stress; intra-NAc shell infusions of either d-amphetamine or opioid agonist DAMGO; GABA agonist, muscimol	Neuroadaptations in NAc shell GABA systems
Pérez-Ortiz (2016) [56]	Animal	C57BL/6J male mice Age: 4 weeks	20/20	High fat diet followed by 12 h food deprivation	Palatable food seeking; expression of potential addiction biomarkers in the NAc: fumarate hydratase (FH), ATP synthase subunit alpha (ATP5a1) and transketolase (TKT)
Pickering (2009) [66] study 1	Animal	Male Wistar rats	12/0	Sugar content of pellets (50% vs. 95%)	Sugar consumption
Pickering (2009) [66] study 2	Animal	Male Wistar rats	9/3	High-fat high-sugar diet (lard, sucrose)	Caloric intake; body weight change
Pickering (2009) [66] study 3	Animal	Male Wistar rats	16/8	High-fat high-sugar diet (lard, sucrose); vulnerability for weight gain; withdrawal from lard and sucrose diet	Caloric intake; body weight change; motivation for sugar; anxiety-like behavior (open-field test)
Schulte (2015) [13] study 1	Cross-sectional	Undergraduates Female: 67.5% Age: 19.3 years BMI: 23.0 kg/m^2^ Caucasian: 72.5%	120/0	Food items (e.g., chocolate, broccoli) and types (e.g., processed)	Food addiction symptoms (YFAS)
Schulte (2015) [13] study 2	Cross-sectional	Adults Male: 59.4% Age: 31.1 years BMI: 27.0 kg/m^2^ Caucasian: 76.8%	384/0	Food characteristics (e.g., proportions of fats, carbohydrates; level of processing)	Self-reported problematic eating behavior
Sharma (2013) [46] study 1	Animal	Male mice Age: 6–7 weeks	12 (treated + control)	High fat diet (58% kcal from fat: including hydrogenated coconut oil, maltodextrin, sucrose, casein) vs. ingredient-matched low fat diet (10.5% kcal from fat); withdrawal from diet	Motivation for sucrose or high-fat reward; caloric intake; body weight change
Sharma (2013) [46] study 2	Animal	Male mice Age: 6–7 weeks	30 (treated + control)	High fat diet vs. low fat diet (10.5% kcal from fat); withdrawal from diet	Anxiety-like behavior (elevated plus maze); plasma corticosterone
Sharma (2013) [46] study 3	Animal	Male mice Age: 6–7 weeks	48 (treated + control)	High fat diet vs. low fat diet (10.5% kcal from fat); withdrawal from diet	Basal corticosterone; protein levels for tyrosine hydroxylase, corticosterone releasing factor type 1 receptor, BDNF, phospho-CREB and ΔFosB in amygdala, NAc and ventral tegmental area via western immunoblotting
Spring (2008) [64]	Double-blind within and between subjects cross-over	Women who are overweight or obese Age: 28.0 years BMI: 27.6 kg/m^2^ Caucasian: 50.8%	61/0	Negative mood; consumption of carbohydrate-rich beverage	Mood; drink preference (carbohydrate-rich vs. macronutrient-balanced) during negative mood state
Tuomisto (1999) [61] study 1	Case-control	Women Age: 35.4 years BMI: 26.7 kg/m^2^	16/15	Self-identified “chocolate addiction”; type of exposure to chocolate (look, smell, or taste)	Psychological symptoms (e.g., depression, disinhibition, disordered eating) measured by questionnaires; self-reported reactivity to cues (e.g., anxiety, calmness); salivation, heart rate
Tuomisto (1999) [61] study 2	Case-control	Women Age: 35.4 years BMI: 26.7 kg/m^2^	16/15	Self-identified “chocolate addiction”; type of exposure to chocolate (look or smell)	Amount of chocolate consumed; psychological symptoms (e.g., depression, disinhibition, disordered eating) measured by questionnaires; self-reported reactivity to cues (e.g., anxiety, calmness); salivation, heart rate
Yakovenko (2011) [67] study 1	Animal	Occidental low- and high-saccharin-consuming rats (LoS and HiS, respectively) Age: 60–90 days	13–15/0	Line of ingestive phenotype (LoS vs. HiS); periodic access to glucose solution followed by 24 h food withdrawal	Withdrawal symptoms (acoustic startle); glucose consumption
Yakovenko (2011) [67] study 2	Animal	LoS and HiS rats Age: 60–90 days	8/0	Line of ingestive phenotype (LoS vs. HiS); periodic access to glucose solution followed by 24 h food withdrawal; naloxone	Withdrawal symptoms (e.g., startle behavior); glucose consumption
Yakovenko (2011) [67] study 3	Animal	LoS and HiS rats Age: 60–90 days	8/0	Binge-like feeding of cookies and shortening	Cookie, shortening, and ethanol consumption

**Table A2:** Risk of bias assessment of included studies.

	Was a Random or Pseudo Random Sample Used?	Was the Inclusion Criteria Clearly Defined?	Were Confounding Factors Identified and Control Strategies Stated?	Were Outcomes Assessed Using Objective Criteria?	Was There Sufficient Description of the Groups?	Is There a Description of Withdrawals and Drop-Outs?	Are the Methods of Statistical Analysis Described?	Is the Source of Financial Support Described?	Is There a Description of Investigators and Assessors, with Possible Conflicts of Interest?	Quality Score [Y/(N + UC)]
Adams (2015) [41]	Y	Y	Y	Y	Y	Y	Y	Y	Y	All Ys
Burmeister (2013) [62]	N	Y	UC	Y	N/A	Y	Y	N	N	1
Cambridge (2013) [40]	Y	Y	Y	Y	Y	Y	Y	Y	Y	All Ys
Colantuoni (2001) [47]	Y	Y	Y	Y	Y	Y	Y	Y	N	8
Cornelis (2016) [69]	N	Y	Y	Y	N/A	Y	Y	Y	Y	7
Daubenmier (2014) [53]	Y	Y	Y	Y	UC	Y	Y	Y	N	3.5
Davis (2011) [39]	N	Y	UC	Y	Y	UC	Y	N	N	0.8
Davis (2013) [49]	N	Y	Y	Y	Y	UC	Y	Y	Y	3.5
Davis (2014) [50]	Y	Y	Y	Y	Y	N	Y	N	Y	3.5
De Ridder (2016) [58]	N	Y	Y	Y	Y	UC	Y	N	Y	2
Duarte (2014) [54]	Y	Y	Y	Y	Y	Y	Y	Y	N	8
Feldstein Ewing (2017) [59]	N	Y	Y	Y	N/A	N	Y	Y	Y	3
Fowler (2014) [70]	UC	Y	Y	Y	UC	N	UC	Y	Y	1.25
Franken (2016) [60]	N	Y	Y	Y	Y	Y	Y	N	Y	3.5
Furlong (2014) [42]	Y	Y	Y	Y	Y	Y	Y	Y	Y	All Ys
Gearhardt (2011) [12]	UC	Y	Y	Y	N	N	Y	Y	Y	2
Imperatori (2015) [57]	UC	Y	Y	Y	Y	UC	Y	N	Y	2
Johnson (2010) [48]	Y	Y	Y	Y	Y	UC	Y	Y	Y	8
Konkolÿ Thege (2015) [5]	N	N	UC	Y	Y	Y	Y	Y	Y	2
Le Merrer (2006) [51]	Y	Y	Y	Y	Y	N	Y	N	N	2
Lenoir (2007) [43]	Y	Y	Y	Y	Y	Y	Y	Y	Y	All Ys
Lent (2012) [63]	N	N	Y	Y	N/A	Y	Y	Y	Y	3
Mangabeira (2015) [65]	Y	Y	Y	Y	Y	UC	Y	Y	N	3.5
Markus (2017) [44]	N	N	Y	Y	N	UC	Y	Y	Y	1.25
Mary Brown (2015) [55]	Y	Y	Y	Y	Y	UC	Y	Y	Y	8
McGee (2010) [68]	Y	Y	Y	Y	Y	Y	Y	Y	N	8
Merlo (2009) [18]	N	Y	UC	Y	Y	UC	Y	Y	N	1.25
Newman (2013) [52]	Y	Y	Y	Y	Y	UC	Y	Y	Y	8
Pérez-Ortiz (2016) [56]	Y	Y	Y	Y	Y	Y	Y	Y	N	8
Pickering (2009) [66]	N	Y	Y	Y	Y	Y	Y	Y	N	3.5
Schulte (2015) [13]	N	N	Y	Y	N/A	Y	Y	Y	Y	3
Sharma (2013) [46]	Y	Y	Y	Y	Y	UC	Y	Y	Y	8
Spring (2008) [64]	N	Y	Y	N	N/A	Y	Y	Y	N	1.67
Tuomisto (1999) [61]	Y	UC	Y	Y	Y	Y	Y	N	N	2
Yakovenko (2011) [67]	Y	Y	Y	Y	Y	UC	Y	Y	N	3.5

N = No, Y = Yes, UC = Unclear, N/A = Non-Applicable.

**Table A3:** Evidence for and against addiction criteria.

Characteristic	Supporting Evidence	Null/Contrary Evidence
Brain changes	Animal studies: Adams (2015) [41] Colantuoni (2001) [47] Duarte (2014) [54] Furlong (2014) [42] study 2 Johnson (2010) [48] study 2 Johnson (2010) [48] study 4 Le Merrer (2006) [51] study 3 Le Merrer (2006) [51] study 4 Mary Brown (2015) [55] Newman (2013) [52] Pérez-Ortiz (2016) [56] Sharma (2013) [46] study 3 Human studies: 13. Cambridge (2013) [40] 14. Daubenmier (2014) [53] 15. Davis (2013) [49] 16. Davis (2014) [50] 17. De Ridder (2016) [58] 18. Feldstein Ewing (2017) [59] 19. Franken (2016) [60] 20. Gearhardt (2011) [12] 21. Imperatori (2015) [57]	Adams (2015) [41] (animal study) Feldstein Ewing (2017) [59] (human study)
Preoccupation	Human studies: Merlo (2009) [18]. Tuomisto (1999) [61] study 1. Tuomisto (1999) [61] study 2.	N/A
Impaired control	Animal studies: Furlong (2014) [42] study 1—Spending a significant amount of time acquiring, using, or recovering from a substance. Lenoir (2007) [43]—Craving. Mary Brown (2015) [55]—Spending a significant amount of time acquiring, using, or recovering from a substance. Human studies: 4. Burmeister (2013) [62]—Consuming a substance in greater amounts or over longer periods of time than intended. 5. Davis (2011) [39]—Craving. 6. Davis (2013) [49]—Craving. 7. Davis (2014) [50]—Craving. 8. Feldstein Ewing (2017) [59]—Craving. 9. Lent (2012) [63]—Craving. 10. Merlo (2009) [18]—Consuming a substance in greater amounts or over longer periods of time than intended. 11. Tuomisto (1999) [61] study 1—Craving. 12. Tuomisto (1999) [61] study 2—Craving.	N/A
Social impairment	Animal studies: Adams (2015) [41]—Continually using a substance despite its effects causing or exacerbating persistent or recurrent social or interpersonal problems. Human studies: 2. Lent (2012) [63]—Giving up or reducing social, occupational, or recreational activities.	N/A
Risky use	Animal studies: Johnson (2010) [48] study 3—Continually using a substance in situations in which it is physically dangerous.	N/A
Tolerance/Withdrawal	Animal studies: Johnson (2010) [48] study 1—Tolerance. Mangabeira (2015) [65]—Withdrawal. Pickering (2009) [66] study 3—Withdrawal. Sharma (2013) [46] study 2—Withdrawal. Human studies: 5. De Ridder (2016) [58]—Withdrawal. 6. Lent (2012) [63]—Tolerance; Withdrawal. 7. Markus (2017) [44]—Tolerance; Withdrawal. 8. Spring (2008) [64]—Tolerance.	Yakovenko (2011) [67] study 1 Yakovenko (2011) [67] study 2 (animal studies)
Chronicity	Animal studies: McGee (2010) [68]. Pickering (2009) [66] study 3.	Konkolÿ Thege (2015) [5] (human study)
Relapse	Animal studies: Pickering (2009) [66] study 3. Sharma (2013) [46] study 1.	N/A
Overall	Human studies: Tuomisto (1999) [61] study 1. Tuomisto (1999) [61] study 2. Schulte (2015) [13] study 1. Schulte (2015) [13] study 2.	N/A

## References

[1] American Psychiatric Association (2013). Diagnostic and Statistical Manual of Mental Disorders.

[2] Carter A., Hendrikse J., Lee N., Yucel M., Verdejo-Garcia A., Andrews Z., Hall W. (2016). The Neurobiology of “Food Addiction” and Its Implications for Obesity Treatment and Policy. Annu. Rev. Nutr..

[3] Ruffle J.K. (2014). Molecular neurobiology of addiction: What’s all the (Δ) FosB about?. Am. J. Drug Alcohol Abus..

[4] Volkow N.D., Wang G.J., Fowler J.S., Tomasi D. (2012). Addiction circuitry in the human brain. Annu. Rev. Pharmacol. Toxicol..

[5] Konkolÿ Thege B., Woodin E.M., Hodgins D.C., Williams R.J. (2015). Natural course of behavioral addictions: A 5-year longitudinal study. BMC Psychiatry.

[6] Alavi S.S., Ferdosi M., Jannatifard F., Eslami M., Alaghemandan H., Setare M. (2012). Behavioral addiction versus substance addiction: Correspondence of psychiatric and psychological views. Int. J. Prev. Med..

[7] Olsen C.M. (2012). Natural rewards, neuroplasticity, and non-drug addictions. Neuropharmacology.

[8] Moreno C., Tandon R. (2011). Should overeating and obesity be classified as an addictive disorder in DSM-5?. Curr. Pharm. Des..

[9] Potenza M.N. (2014). Non-substance addictive behaviors in the context of DSM-5. Addict. Behav..

[10] American Society of Addiction Medicine. http://www.asam.org/quality-practice/definition-of-addiction.

[11] Davis C., Carter J.C. (2009). Compulsive overeating as an addiction disorder. A review of theory and evidence. Appetite.

[12] Gearhardt A.N., Yokum S., Orr P.T., Stice E., Corbin W.R., Brownell K.D. (2011). Neural correlates of food addiction. Arch. Gen. Psychiatry.

[13] Schulte E.M., Avena N.M., Gearhardt A.N. (2015). Which foods may be addictive? The roles of processing, fat content, and glycemic load. PLoS ONE.

[14] Meule A., Gearhardt A.N. (2014). Food addiction in the light of DSM-5. Nutrients.

[15] English Oxford Living Dictionaries. https://en.oxforddictionaries.com/definition/addicted.

[16] American Psychological Association. http://www.apa.org/topics/addiction/.

[17] American Psychiatric Association. https://www.psychiatry.org/patients-families/addiction/what-is-addiction.

[18] Merlo L.J., Klingman C., Malasanos T.H., Silverstein J.H. (2009). Exploration of food addiction in pediatric patients: A preliminary investigation. J. Addict. Med..

[19] Ifland J.R., Preuss H.G., Marcus M.T., Rourke K.M., Taylor W.C., Burau K., Jacobs W.S., Kadish W., Manso G. (2009). Refined food addiction: A classic substance use disorder. Med. Hypotheses.

[20] Gearhardt A.N., Corbin W.R., Brownell K.D. (2009). Preliminary validation of the Yale food addiction scale. Appetite.

[21] Gearhardt A.N., Corbin W.R., Brownell K.D. (2016). Development of the Yale Food Addiction Scale Version 2.0. Psychol. Addict. Behav..

[22] Benton D., Young H.A. (2016). A meta-analysis of the relationship between brain dopamine receptors and obesity: A matter of changes in behavior rather than food addiction?. Int. J. Obes..

[23] Riva G., Bacchetta M., Cesa G., Conti S., Castelnuovo G., Mantovani F., Molinari E. (2006). Is severe obesity a form of addiction? Rationale, clinical approach, and controlled clinical trial. Cyberpsychol. Behav..

[24] Volkow N.D., Wang G.J., Tomasi D., Baler R.D. (2013). The addictive dimensionality of obesity. Biol. Psychiatry.

[25] Ziauddeen H., Farooqi I.S., Fletcher P.C. (2012). Obesity and the brain: How convincing is the addiction model?. Nat. Rev. Neurosci..

[26] Burrows T., Skinner J., McKenna R., Rollo M. (2017). Food Addiction, Binge Eating Disorder, and Obesity: Is There a Relationship?. Behav. Sci..

[27] Davis C. (2017). A commentary on the associations among ‘food addiction’, binge eating disorder, and obesity: Overlapping conditions with idiosyncratic clinical features. Appetite.

[28] Pursey K.M., Stanwell P., Gearhardt A.N., Collins C.E., Burrows T.L. (2014). The prevalence of food addiction as assessed by the Yale Food Addiction Scale: A systematic review. Nutrients.

[29] Long C.G., Blundell J.E., Finlayson G. (2015). A systematic review of the application and correlates of YFAS-diagnosed ‘food addiction’ in humans: Are eating-related ‘addictions’ a cause for concern or empty concepts?. Obes. Facts.

[30] Corwin R.L., Hayes J.E., Rippe J.M. (2014). Are sugars addictive? Perspectives for practitioners. Fructose, High Fructose Corn Syrup, Sucrose and Health.

[31] Hone-Blanchet A., Fecteau S. (2014). Overlap of food addiction and substance use disorders definitions: Analysis of animal and human studies. Neuropharmacology.

[32] Ruddock H.K., Dickson J.M., Field M., Hardman C.A. (2015). Eating to live or living to eat? Exploring the causal attributions of self-perceived food addiction. Appetite.

[33] Wallace D.L., Vialou V., Rios L., Carle-Florence T.L., Chakravarty S., Kumar A., Graham D.L., Green T.A., Kirk A., Iñiguez S.D. (2008). The influence of DeltaFosB in the nucleus accumbens on natural reward-related behavior. J. Neurosci..

[34] Pursey K.M., Davis C., Burrows T.L. (2017). Nutritional Aspects of Food Addiction. Curr. Addict. Rep..

[35] Liberati A., Altman D.G., Tetzlaff J., Mulrow C., Gøtzsche P.C., Ioannidis J.P., Clarke M., Devereaux P.J., Kleijnen J., Moher D. (2009). The PRISMA statement for reporting systematic reviews and meta-analyses of studies that evaluate health care interventions: Explanation and elaboration. J. Clin. Epidemiol..

[36] Moher D., Liberati A., Tetzlaff J., Altman D.G. (2009). Preferred reporting items for systematic reviews and meta-analyses: The PRISMA statement. Ann. Intern. Med..

[37] American Psychological Association. http://www.apa.org/pubs/databases/training/method-values.aspx.

[38] Jamaty C., Bailey B., Larocque A., Notebaert E., Sanogo K., Chauny J.M. (2010). Lipid emulsions in the treatment of acute poisoning: A systematic review of human and animal studies. Clin. Toxicol..

[39] Davis C., Curtis C., Levitan R.D., Carter J.C., Kaplan A.S., Kennedy J.L. (2011). Evidence that ‘food addiction’ is a valid phenotype of obesity. Appetite.

[40] Cambridge V.C., Ziauddeen H., Nathan P.J., Subramaniam N., Dodds C., Chamberlain S.R., Koch A., Maltby K., Skeggs A.L. (2013). Napolitano, A. Neural and behavioral effects of a novel mu opioid receptor antagonist in binge-eating obese people. Biol. Psychiatry.

[41] Adams W.K., Sussman J.L., Kaur S., D’Souza A.M., Kieffer T.J., Winstanley C.A. (2015). Long-term, calorie-restricted intake of a high-fat diet in rats reduces impulse control and ventral striatal D_2_ receptor signaling—Two markers of addiction vulnerability. Eur. J. Neurosci..

[42] Furlong T.M., Jayaweera H.K., Balleine B.W., Corbit L.H. (2014). Binge-like consumption of a palatable food accelerates habitual control of behavior and is dependent on activation of the dorsolateral striatum. J. Neurosci..

[43] Lenoir M., Serre F., Cantin L., Ahmed S.H. (2007). Intense sweetness surpasses cocaine reward. PLoS ONE.

[44] Markus C.R., Rogers P.J., Brouns F., Schepers R. (2017). Eating dependence and weight gain; no human evidence for a ‘sugar-addiction’ model of overweight. Appetite.

[45] Chartres N., Fabbri A., Bero L.A. (2016). Association of industry sponsorship with outcomes of nutrition studies: A systematic review and meta-analysis. JAMA Intern. Med..

[46] Sharma S., Fernandes M.F., Fulton S. (2013). Adaptations in brain reward circuitry underlie palatable food cravings and anxiety induced by high-fat diet withdrawal. Int. J. Obes..

[47] Colantuoni C., Schwenker J., McCarthy J., Rada P., Ladenheim B., Cadet J.L., Schwartz G.J., Moran T.H., Hoebel B.G. (2001). Excessive sugar intake alters binding to dopamine and mu-opioid receptors in the brain. Neuroreport.

[48] Johnson P.M., Kenny P.J. (2010). Dopamine D2 receptors in addiction-like reward dysfunction and compulsive eating in obese rats. Nat. Neurosci..

[49] Davis C., Loxton N.J., Levitan R.D., Kaplan A.S., Carter J.C., Kennedy J.L. (2013). ‘Food addiction’ and its association with a dopaminergic multilocus genetic profile. Physiol. Behav..

[50] Davis C., Levitan R.D., Kaplan A.S., Kennedy J.L., Carter J.C. (2014). Food cravings, appetite, and snack-food consumption in response to a psychomotor stimulant drug: The moderating effect of ‘food-addiction’. Front. Psychol..

[51] Le Merrer J., Stephens D.N. (2006). Food-induced behavioral sensitization, its cross-sensitization to cocaine and morphine, pharmacological blockade, and effect on food intake. J. Neurosci..

[52] Newman S., Pascal L., Sadeghian K., Baldo B.A. (2013). Sweetened-fat intake sensitizes gamma-aminobutyric acid—Mediated feeding responses elicited from the nucleus accumbens shell. Biol. Psychiatry.

[53] Daubenmier J., Lustig R.H., Hecht F.M., Kristeller J., Woolley J., Adam T., Dallman M., Epel E. (2014). A new biomarker of hedonic eating? A preliminary investigation of cortisol and nausea responses to acute opioid blockade. Appetite.

[54] Duarte R.B.M., Patrono E., Borges A.C., César A.A.S., Tomaz C., Ventura R., Gasbarri A., Puglisi-Allegra S., Barros M. (2014). Consumption of a highly palatable food induces a lasting place-conditioning memory in marmoset monkeys. Behav. Process..

[55] Mary Brown R., Michael Kupchik Y., Spencer S., Garcia-Keller C., Spanswick D.C., John Lawrence A., Jhou T., William Kalivas P. (2015). Addiction-like synaptic impairments in diet-induced obesity. Bio. Psychiatry.

[56] Pérez-Ortiz J.M., Galiana-Simal A., Salas E., González-Martín C., García-Rojo M., Alguacil L.F. (2016). A high-fat diet combined with food deprivation increases food seeking and the expression of candidate biomarkers of addiction. Addict. Biol..

[57] Imperatori C., Fabbricatore M., Innamorati M., Farina B., Quintiliani M.I., Lamis D.A., Mazzucchi E., Contardi A., Vollono C., Marca G.D. (2015). Modification of EEG functional connectivity and EEG power spectra in overweight and obese patients with food addiction: An eLORETA study. Brain Imaging Behav..

[58] De Ridder D., Manning P., Leon S.L., Ross S., Sutherland W., Horwath C., Vanneste S. (2016). The brain, obesity and addiction: An EEG neuroimaging study. Sci. Rep..

[59] Feldstein Ewing S.W., Claus E.D., Hudson K.A., Filbey F.M., Jimenez E.Y., Lisdahl K.M., Kong A.S. (2017). Overweight adolescents’ brain response to sweetened beverages mirrors addiction pathways. Brain Imaging Behav..

[60] Franken I.H., Nijs I.M., Toes A., van der Veen F.M. (2016). Food addiction is associated with impaired performance monitoring. Biol. Pyschol..

[61] Tuomisto T., Hetherington M.M., Morris M.F., Tuomisto M., Turjanmaa V., Lappalainen R. (1999). Psychological and physiological characteristics of sweet food ‘addiction’. Int. J. Eat. Disord..

[62] Burmeister J.M., Hinman N., Koball A., Hoffmann D.A., Carels R.A. (2013). Food addiction in adults seeking weight loss treatment. Implications for psychosocial health and weight loss. Appetite.

[63] Lent M.R., Swencionis C. (2012). Addictive personality and maladaptive eating behaviors in adults seeking bariatric surgery. Eat. Behav..

[64] Spring B., Schneider K., Smith M., Kendzor D., Appelhans B., Hedeker D., Pagoto S. (2008). Abuse potential of carbohydrates for overweight carbohydrate cravers. Psychopharmacology.

[65] Mangabeira V., Garcia-Mijares M., Silva M.T. (2015). Sugar withdrawal and differential reinforcement of low rate (DRL) performance in rats. Physiol. Behav..

[66] Pickering C., Alsiö J., Hulting A.L., Schiöth H.B. (2009). Withdrawal from free-choice high-fat high-sugar diet induces craving only in obesity-prone animals. Psychopharmacology.

[67] Yakovenko V., Speidel E.R., Chapman C.D., Dess N.K. (2011). Food dependence in rats selectively bred for low versus high saccharin intake. Implications for ‘food addiction’. Appetite.

[68] McGee H.M., Amare B., Bennett A.L., Duncan-Vaidya E.A. (2010). Behavioral effects of withdrawal from sweetened vegetable shortening in rats. Brain Res..

[69] Cornelis M.C., Flint A., Field A.E., Kraft P., Han J., Rimm E.B., van Dam R.M. (2016). A genome-wide investigation of food addiction. Obesity.

[70] Fowler L., Ivezaj V., Saules K.K. (2014). Problematic intake of high-sugar/low-fat and high glycemic index foods by bariatric patients is associated with development of post-surgical new onset substance use disorders. Eat. Behav..

[71] Hebebrand J., Albayrak Ö., Adan R., Antel J., Dieguez C., de Jong J., Leng G., Menzies J., Mercer J.G., Murphy M. (2014). “Eating addiction”, rather than “food addiction”, better captures addictive-like eating behavior. Neurosci. Biobehav. Rev..

[72] Ifland J., Preuss H.G., Marcus M.T., Rourke K.M., Taylor W., Wright H.T. (2015). Clearing the confusion around processed food addiction. J. Am. Coll. Nutr..

[73] Lustig R.H., Schmidt L.A., Brindis C.D. (2012). Public health: The toxic truth about sugar. Nature.

[74] Nolan L.J. (2017). Is it time to consider the “food use disorder”?. Appetite.

[75] Schulte E.M., Potenza M.N., Gearhardt A.N. (2016). A commentary on the “eating addiction” versus “food addiction” perspectives on addictive-like food consumption. Appetite.

[76] National Institute on Drug Abuse Tobacco/Nicotine. https://www.drugabuse.gov/publications/research-reports/tobacconicotine.

[77] Avena N.M., Rada P., Hoebel B.G. (2008). Evidence for sugar addiction: Behavioral and neurochemical effects of intermittent, excessive sugar intake. Neurosci. Biobehav. Rev..

[78] Černý L., Černý K. (1992). Can carrots be addictive? An extraordinary form of drug dependence. Br. J. Addict..

[79] Pedram P., Sun G. (2014). Hormonal and dietary characteristics in obese human subjects with and without food addiction. Nutrients.

[80] Curtis C., Davis C. (2014). A qualitative study of binge eating and obesity from an addiction perspective. Eat. Disord..

